# Directional Deep Brain Stimulation Lead Rotation in the Early Postoperative Period

**DOI:** 10.1227/neuprac.0000000000000102

**Published:** 2024-07-19

**Authors:** Huy Q. Dang, Gabriel Reyes, Ethan Devara, Nisha Giridharan, Anthony K. Allam, Garrett P. Banks, Ashwin Viswanathan, Ben Shofty, Sameer A. Sheth

**Affiliations:** * Department of Neurosurgery, Baylor College of Medicine, Houston, Texas, USA;; ‡ Department of Neurosurgery, University of Utah, Salt Lake City, Utah, USA

**Keywords:** Lead rotation, DBS, Accuracy, Directional

## Abstract

**BACKGROUND AND OBJECTIVES:**

Directional deep brain stimulation (DBS) enables treatment optimization by current steering using segmented leads. Identification of the lead's rotational orientation is critical to guide programming decisions. Orientation is often assessed during or immediately after implant, but the degree of lead rotation in the following weeks is not well appreciated. Our objective was to measure the degree of DBS lead rotational orientation changes within the first few weeks after surgery.

**METHODS:**

We retrospectively reviewed the clinical records of patients who were implanted with segmented DBS leads at our institution. All included patients had at least 1 immediate postoperative computed tomography (CT) (CT1) and another CT at least 1 week later (CT2). We assessed lead rotational orientation angles on CT1 and CT2 and calculated the degrees of rotation change between the scans. We also assessed for any effect of the time interval between scans by calculating the correlation between CT1-CT2 latency and degrees of lead rotation.

**RESULTS:**

We assessed a total of 75 DBS lead orientations for 38 patients. The average change in lead orientation between CT1 and CT2 was 8.6° (median = 2.9°, range = 0.11-168.2°). Only 8 percent of patients (3/38) were found to have a significant change in orientation (>30°); however, when it occurred, it occurred bilaterally. There was no correlation between CT1-CT2 latency and lead rotation (r(74) = 0.04, *P* = .73).

**CONCLUSION:**

Our study finds that changes in lead orientation occurring over the first few weeks after surgery are rare. Thus, for most patients, the immediate postoperative CT is adequate for determining the orientation angles for clinical programming. However, if programming is found to be difficult, a repeat CT scan could be beneficial for a minority of patients.

**ABBREVIATION:**
DiODe: directional orientational detection.

The efficacy of deep brain stimulation (DBS) is contingent upon accurate lead placement because most target nuclei are small and surrounded by critical structures.^1^ Improvements in stereotactic procedures as well as electrophysiological recordings and advanced imaging methods have progressively reduced targeting error and helped optimize therapeutic effects.^2^ DBS leads with segmented electrodes allow clinicians to further fine tune stimulation volume shapes to better target regions of beneficial effect and avoid regions causing adverse effects.^3^ Currently, all 3 commercially available DBS systems in the United States have options for segmented leads with directional tuning capabilities. These segmented leads typically have 4 electrode levels, similar to the traditional quadripolar lead, but the 2 middle electrode levels contain 3 equally spaced contact segments.^4^ The distribution of charge across these contacts (especially the segmented ones) determines the shape of the electric field. Compared with ring electrodes which can only activate a toroidal volume of tissue around the lead, segmented electrodes can create electric fields that are biased in an intended direction to increase the therapeutic window.^5^

Directionally tuned stimulation can increase the therapeutic potential by allowing greater customization of programming configurations.^6^ However, the stability of lead orientation after placement is critical to ensure that directional stimulation is being aimed toward the intended target. Lead orientation is typically evaluated on either an intraoperative or immediate postoperative computed tomography (CT). Changes in lead orientation after implant would obviate that initial measurement and, if not accounted for, misinform subsequent programming. Effects that could cause this change include rotational slippage of the lead in the burr hole cover (possibly because of angular torque produced when tunneling the lead) or resolution of brain shift secondary to pneumocephalus, if present, after the implant surgery.

In this study, we assessed lead orientation on the immediate postoperative CT and compared it with the lead orientation seen on the CT obtained 1 to 3 weeks later with the goal of determining the extent to which leads rotate in the first few weeks after implant.

## METHODS

### Clinical Data

We performed a retrospective analysis of patients who underwent DBS surgery at our institution between 2018 and 2023. The study falls under our departmental Institutional Review Board for retrospective review of clinically acquired data. Patient consent was not required because of the retrospective nature of this study.

Patients were included in this study if they were implanted with Boston Scientific segmented DBS leads (Vercise Cartesia™ Directional System, Model DB 2202-45, Boston Scientific). We limited our analysis to only these leads because of their compatibility with the lead detection algorithm used in our study. We also only included patients if they had more than 1 eligible postoperative CT for comparison. At a minimum, patients needed to have an immediate postoperative CT and another CT performed at least 1 week after surgery. Eligible CTs were acquired with a stereotactic protocol appropriate for automated lead detection (1 mm or thinner axial cut CT scans).

All patients received preoperative MRI and CT scans for stereotactic planning. Two surgeons at our institution performed the DBS surgeries using robot-assisted stereotaxy (ROSA ONE Brain, Zimmer Biomet). The technique used has been previously described in the study by Giridharan et al, 2022.^7^ An immediate postoperative CT (CT1) was performed within 2 to 8 hours after surgery as part of a routine assessment for lead placement. A subsequent CT (CT2) was acquired at least 1 week later, usually at the time of the admission for neurostimulator implant. It is our standard practice to acquire these delayed CTs, once brain shift from reasons such as pneumocephalus has resolved. This delayed CT scan is used by the neurology team to determine the final lead location for image-based programming. Thus, CT2 is a clinically acquired scan, not obtained just for research purposes.

### Lead Position Measurements

**FIGURE 1.**
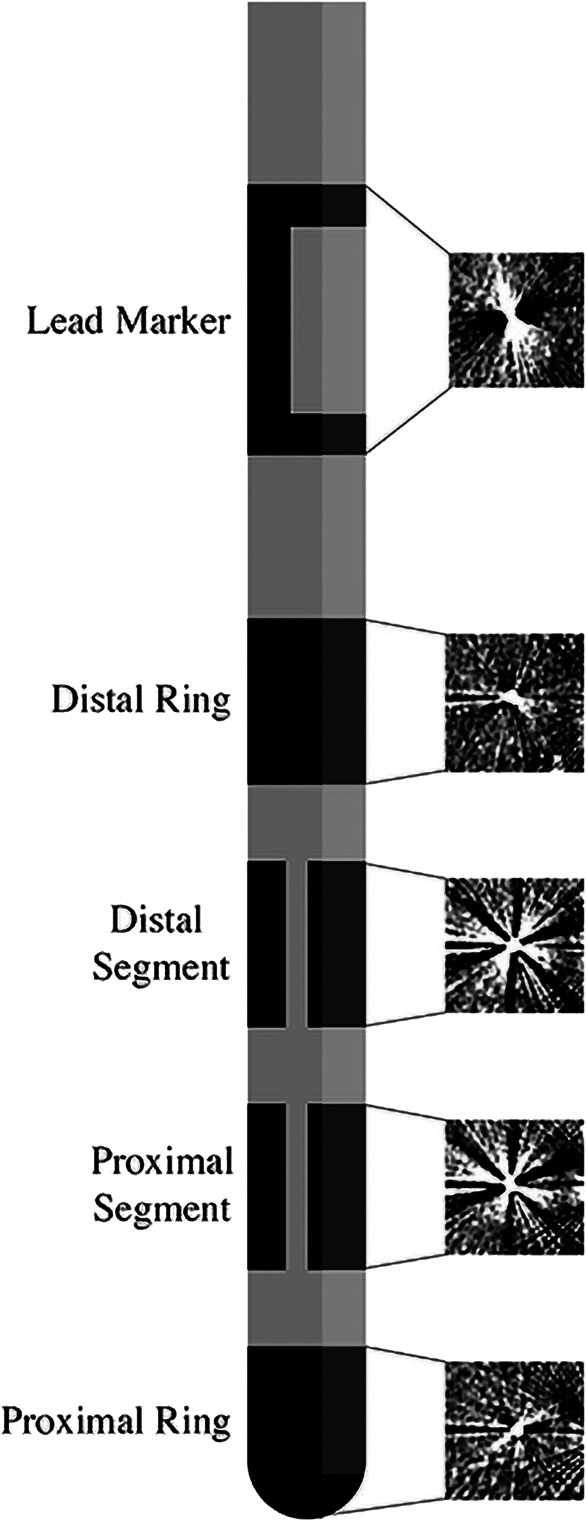
Illustration of segmented lead and scatter artifacts at different levels on CT. Scatter artifact patterns seen on CT created by the electrodes at different levels. These patterns are recognized by the directional orientational detection algorithm to estimate lead orientation. CT, computed tomography.

**FIGURE 2.**
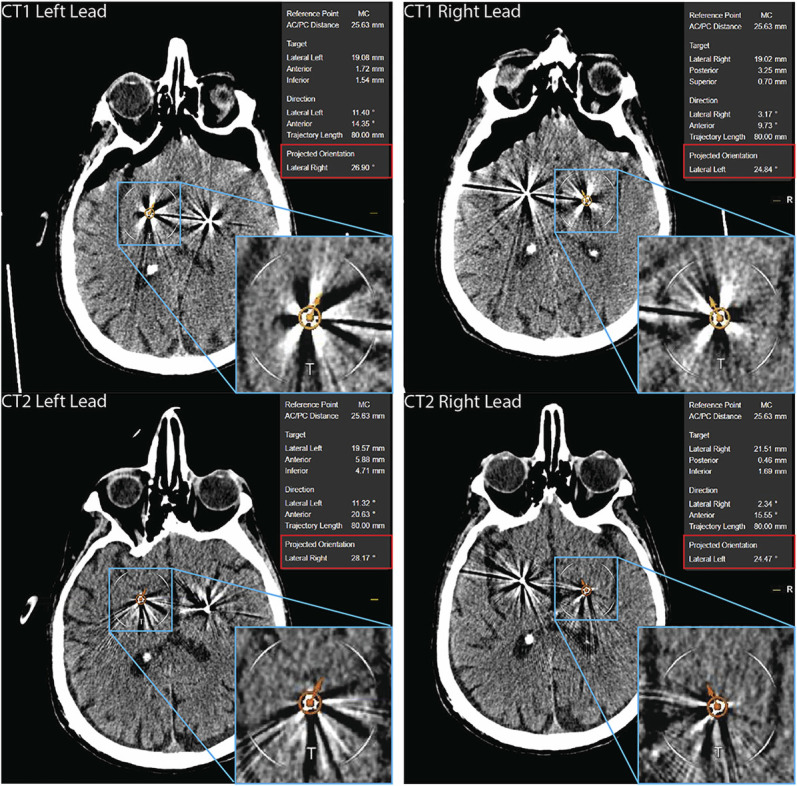
Orientation angles found on CT1 and CT2 based on the DiODe algorithm. Automatic lead detection using the Brainlab software's built-in DiODe algorithm provides an estimation of the lead orientation for the anterior commissure/posterior commissure line. Orientation of the left lead in CT1 (yellow arrows) and CT2 (red arrows) is displayed on the left panels. Orientation of the right lead in CT1 and CT2 is displayed on the right panels. Embedded images highlight the estimated orientation of the leads. “Projected orientation” (in the red box) is the value of the estimation. Lead rotation is calculated by subtracting the projected orientation angles found on CT1 and CT2. CT, computed tomography; DiODe, directional orientational detection.

We loaded all preoperative T1-weighted MRI images and postoperative CT images into the Brainlab Elements software (Brainlab). We fused the preoperative MRI with CT1 and CT2 and marked the anterior commissure/posterior commissure coordinates as reference points for lead orientation. In Brainlab's Lead Localization workspace, we used the “Detect” tool to automatically identify the tip and axis of implanted leads. After we assigned the “Directional Lead” model, the software automatically estimated the orientation angles based on the Directional Orientation Detection (DiODe) algorithm. The DiODe algorithm recognizes characteristic electrode scatter artifact patterns on CT (Figure 1).^8,9^ Lange et al^10^ found that lead angles estimated using the DiODe algorithm correlated with other established techniques such as rotational fluoroscopy. The reference point is defined by the algorithm as parallel to the anterior commissure/posterior commissure line such that the orientation angle is assigned 0° if the lead marker is parallel and facing anteriorly. The orientation angle is assigned a positive number for a clockwise orientation (as viewed from above; up to +180°) and a negative number for a counterclockwise orientation (up to −179.9°). Lead rotation was calculated as an absolute value of the difference between the estimated orientation angles on CT1 and CT2 (Figure 2). Summary statistics (mean, median, and range) were calculated for each measure. We also calculated the incidence of lead rotation greater than 30°, which we hypothesized may be sufficient to affect the efficacy of image-guided programming.

### Statistical Analysis

We performed statistical analysis using Matlab (MathWorks). We compared the lead orientation angle in CT1 with the lead orientation angle in CT2 of all patients using the nonparametric Wilcoxon signed-rank test to assess for systematic changes. We then compared lead rotation between the left and right hemispheres using the Mann–Whitney *U* test. Finally, we calculated a Spearman correlation coefficient between lead rotation and CT1-CT2 latency, defined as days between CT scans. A *P*-value of <.05 was considered statistically significant.

## RESULTS

Thirty-eight sequential patients (mean age = 65.5 years, SD = 10.5, range = 36-85 years) who were implanted with Boston Scientific segmented leads were included in this study. All but 1 patient had bilateral electrode implants, resulting in a total of 75 leads. Indications for DBS included Parkinson's disease (n = 30) and essential tremor (n = 8). Targets were the subthalamic nucleus (n = 16), globus pallidus internus (n = 13), and ventral intermediate nucleus of the thalamus (n = 9). Each patient underwent 2 CT scans, early postoperative and late, as described in the Methods section. The mean number of days between CT scans was 33 days (SD = 126, median = 14 days, range = 5-789 days).

For each patient, the lead orientation angle on CT1 and CT2 and the degrees of rotation between CT1 and CT2 are summarized in Table. The average lead orientation angle in CT1 for all 75 leads was +11° (SD = 50.1, median = +3.5°, range = −103.1 to +146.3°), and that in CT2 was +16.0° (SD = 47.8, median = +5.6°, range = −88 to +142.8°). These data suggest that there is a nonsignificant clockwise trend, and despite the large range, the average orientation is approximately forward. In addition, when comparing the orientation angles between CT1 and CT2, there was no trend toward rotation in 1 direction over another (*P* = .13, CI [−0.43, 10.30]). The lead orientation angles were also not significantly different between the left and right leads for CT1 (*P* = .56, CI [−34.98, 11.66]) or CT2 (*P* = .75, CI [−38.18, 6.92], Mann–Whitney *U* test).

**TABLE.** Clinical Summary and Lead Orientation and Rotation Statistics

Characteristics	Total
**Surgical indications**	
Parkinson's disease	30
Essential tremor	8
**Targets**	
Subthalamic nucleus	16
Globus pallidus internus	13
Ventral intermediate nucleus of the thalamus	9

Characteristics	Mean ± SD	Median	Range	*P*-value
Days between CT-1 and CT-2 (days)	33 ± 126	126	5-789	
**Orientation angles (°)**				
CT-1 total orientation angles	+11 ± 50.1	+3.5	−103.1, 146.3	.60
CT-2 total orientation angles	+16 ± 47.8	+5.6	−88, 142.8	
Left lead orientation CT-1	4.1 ± 54.4	0.5	−103.1, 146.27	.56
Right lead orientation CT-1	17.8 ± 45.3	10.7	−55.9, 119.5	
Left lead orientation CT-2	8.2 ± 50.9	−0.6	−88, 142.8	.75
Right lead orientation CT-2	23.6 ± 43.8	21.4	−44.1, 118.6	
**Rotation (°)**				
Total rotation	8.6 ± 22.4	2.9	0.11-168.2	
Left lead rotation	8.0 ± 15.0	3.2	0.21-64.8	.33
Right lead rotation	9.3 ± 28.2	2.8	0.11-168.2	

CT, computed tomography.

The mean lead rotation between CT1 and CT2 was 8.6° (SD = 22.4°, median = 2.9°, range = 0.11-168.2°). Of the 75 analyzable leads, 69 leads had less than 30° rotation, whereas 6 leads had more than 30° rotation. Notably, when 1 lead had more than 30 degrees of rotation, the bilateral lead also had more than 30° as well. The 6 leads with greater than 30° occurred bilaterally in 3 patients. The degree of lead rotation was not found to be significantly different between left and right leads (*P* = .33, CI [−0.56, 1.51], Mann–Whitney *U* test). There was also no correlation between CT1-CT2 latency and lead rotation (r(74) = 0.04, *P* = .73, CI [−0.19, 0.26]).

## DISCUSSION

Segmented leads allow clinicians more precise control of the electric field in DBS therapy by enabling rotational current steering in the plane transverse to the lead. However, this precision requires that the clinicians know the orientation of the segmented leads, which is facilitated if the leads maintain a stable position over time. In this study, we analyzed lead rotation in a cohort of patients who were implanted with segmented leads and found that there is infrequently a change in lead orientation after initial lead placement.

Surgical technique during lead implantation may theoretically cause changes in the intended position of segmented contacts. Manual manipulation of the distal end of the lead does not transmit to the tip in a predictable manner because of the flexibility of their construction.^11^ In addition, excess intraoperative lead torsion can result in continuous rotation 24 hours later.^12^ Previous lead rotation studies comparing intraoperative, postfixation lead orientation with immediate (<24 hours) postoperative orientation on CT have shown mixed results. Dembek et al and Lange et al reported large degrees of rotation between the intraoperative lead position and the immediate postoperative lead position,^9,10^ whereas Kruger et al reported no significant rotation.^13^ These contradicting results may be due to different institutional techniques in lead implantation and securing, thus making it difficult to compare. However, all studies agreed that leads typically remain stable beyond the first CT when compared with subsequent CTs.^9,10,13^ We found similar results, demonstrating that leads remain in stable orientation 1 to 3 weeks after being implanted in more than 90% of instances. In general, the immediate postoperative CT adequately accounts for mechanical or torsional forces that may cause intraoperative and perioperative rotations, and thus, routine postoperative imaging is unnecessary for most cases.^14^ One patient in our cohort demonstrated a nearly 180° rotation in their right lead on postoperative day 7; however, their tremor symptoms improved with omnidirectional stimulation, obviating the need to adjust the parameters based on the lead's rotation. For patients who may require more targeted stimulation with directional steering, follow-up imaging may be warranted if a trial with the expected stimulation parameters does not produce the desired effects.

Long-term stability of DBS electrode placement is in part due to the lead securing devices used after implantation, which are designed to anchor the leads.^15^ Once the incisions have been closed, there are few possible sources of torsion on the lead base as it is largely inaccessible. As such, we found no positive correlation between lead rotation and latency between CT scans. Our results indicate that after settling, no significant incremental rotation occurs over time. This would also suggest that the leads remain stable beyond the time constraints of our study. In evaluating long-term stability, Dembek et al conducted a similar study that included patients with longer follow-up CT latency than any other study to date (mean CT-CT latency of 82 days) and still saw no significant differences in lead orientation or clinical effects of rotation months after surgery. The authors performed a linear regression of lead orientation angle vs time and found no relationship between these variables.^16^ The rotation measurements for the 2 patients in our cohort who had repeat CT scans >3 weeks from their operation seem to corroborate these findings because the leads did not experience >30° of rotation. More data with month-year follow-up imaging would be needed to prove definitively that leads do not change over long periods of time.

Taken together, these studies validate the use of an immediate postoperative CT to assess for segmented lead orientation in most patients.^9,10,13^ Clinicians can be reasonably confident that they are using the appropriate electrode segments for directional current steering by analyzing the first postoperative CT. For a majority of cases in which therapy using ring mode either fails or causes side effects, clinicians may switch to segmented mode and avoid these problematic regions without reassessing orientation. However, in cases in which segmented mode (directional steering) is used but does not produce the desired effects or may cause untoward side effects,^17^ repeat imaging may help determine whether extreme lead rotation is a contributing factor. From our analysis, it is found that only a small proportion of the leads had more than 30° of rotation, whereas most of the leads had likely negligible rotation. In addition, imaging for these difficult cases would allow visualization of the relationship between the segmented leads and the neuroanatomy to more precisely shape the electric field away from adverse areas and toward beneficial areas. Titrating directional stimulation for movement disorders may be adequately performed, to a certain extent, by testing several segmented contacts at once in the clinic because the effects of stimulation on motor symptoms are visible and usually have immediate feedback.^17^ By contrast, effects of stimulation for mood disorders are subtler and may take weeks to months to manifest, and thus, precise knowledge of lead orientation and anatomic relationship will be critical to troubleshooting issues with treatment response.^18^ As directional stimulation becomes more widely used for more personalized and precise therapy, the understanding that the first postoperative CT is adequate for estimating lead orientation unless therapy is not working as expected or intolerable side effects occur will become more relevant to clinical decision-making.

### Limitations

A limitation of our study is that it is retrospective, so there is no standardization of CT-CT latency. Our ability to generalize these results to longer timeframes is also limited because almost none of the patients eligible for inclusion in this study received a follow-up CT after the 3-week postoperative period. Finally, we are limited to assessing only Boston Scientific segmented leads because other devices are not compatible with the DiODe algorithm. Thus, nuances in device design may affect lead position but have not yet been characterized.

## CONCLUSION

As DBS technology improves over time, we can more accurately target specific brain regions. Segmented leads are designed to allow clinicians to steer the electric field, theoretically increasing therapeutic flexibility and minimizing side effects. Accounting for possible lead rotation is important to determine the best contacts to use for accurate stimulation targeting and to ensure the stability of therapy over time. Our results indicate that there is no significant rotation of leads within the weeks after initial imaging for the large majority of patients. Thus, we conclude that the lead orientation established on immediate postoperative CT remains stable for most patients and routine reimaging is unlikely necessary for accurate DBS programming.
